# Targeting BCL-XL-mediated apoptotic resistance in lung cancer: *in silico* and *in vitro* identification of bioactive inhibitors from *Ailanthus excelsa* chloroform extract

**DOI:** 10.3389/fmed.2026.1925157

**Published:** 2026-09-09

**Authors:** Abrar Ali, Atul Kumar, Sadaf Anwar, Halima Mustafa Elagib, Farida Habib Khan, Mona M. Shahien, Mohd Adnan Kausar, Mohd Ahamad, Mohammad Salman Akhtar, Kapil Dev, Sheersh Massey

**Affiliations:** 1 Department of Ophthalmology, College of Medicine, University of Ha'il, Hail, Saudi Arabia; 2 Medical Biotechnology Lab, Department of Biotechnology, Jamia Millia Islamia, New Delhi, India; 3 Department of Biochemistry, College of Medicine, University of Ha'il, Hail, Saudi Arabia; 4 Department of Pharmacology, College of Medicine, University of Ha’il, Hail, Saudi Arabia; 5 Department of Community and Family Medicine, College of Medicine, University of Ha'il, Hail, Saudi Arabia; 6 Department of Pediatrics, College of Medicine, University of Ha'il, Hail, Saudi Arabia; 7 Department of Basic Medical Sciences, Faculty of Applied Medical Sciences, Al-Baha University, Al-Baha, Saudi Arabia; 8 Dr. Rajendra Prasad Centre for Ophthalmic Sciences, All India Institute of Medical Sciences, New Delhi, India

**Keywords:** *Ailanthus excelsa*, BCL-2, BCL-XL, decane, 1, 9-bis[(trimethylsilyl)oxy], lung cancer, squalene

## Abstract

**Introduction:**

Lung cancer is one of the most prominent causes of cancer-related death worldwide, primarily driven by apoptosis resistance linked to the overexpression of antiapoptotic proteins. BCL-XL plays a regulatory function in cellular apoptosis, making it a critical therapeutic target in cancer treatment. In this study, we explored the potential therapeutic effects of phytocompounds isolated from leaves of Ailanthus excelsa to inhibit BCL-XL using an integrated approach combining in silico and in vitro methods.

**Methods:**

Phytochemical analysis of the chloroform extract of A. excelsa leaves was performed via gas chromatography-mass spectrometry (GC-MS). Compounds were selected based on their favorable pharmacokinetic properties and subjected to molecular docking against BCL-XL, followed by molecular dynamics simulations over 100 ns, analysis of the simulation trajectory RMSF, principal component analysis, and free-energy landscape. In vitro cytotoxicity of the leaf extract was assessed by MTT assay on lung cancer cell lines.

**Results:**

GC-MS identified 32 major bioactive compounds. Among these, decane,1,9-bis[(trimethylsilyl)oxy] and squalene were selected based on their favorable pharmacokinetic properties. Molecular docking studies revealed that both these compounds interact with the BH3-binding groove of BCL-XL through hydrophobic interactions. Further molecular dynamics simulations of decane,1,9-bis[(trimethylsilyl)oxy] and squalene over 100 ns confirmed the stability and compactness of the protein-ligand complex. The results of the simulation trajectory RMSF indicated stable structural conformations and minor fluctuations around the BH3 domain, suggesting effective binding. Principal component analysis and free-energy landscape exhibited that the binding of squalene promotes multiple conformational states, while decane,1,9-bis[(trimethylsilyl)oxy] limits flexibility in the protein conformations. In addition, the MTT assay conducted on lung cancer cell lines showed significant cytotoxicity of the A. excelsa leaf extract in a dose-dependent manner, highlighting its anticancer properties.

**Discussion:**

This study emphasizes the therapeutic potential of phytocompounds as natural inhibitors of BCL-XL. The integration of GC-MS profiling, molecular docking, molecular dynamics simulations, and in vitro studies provides a comprehensive strategy for the identification of plant-derived lead molecules targeting critical cancer pathways. Further in vivo investigations and molecular validations would provide better insights into BCL-XL-targeted Phyto-therapeutics for lung cancer.

## Introduction

**Figure 1:**
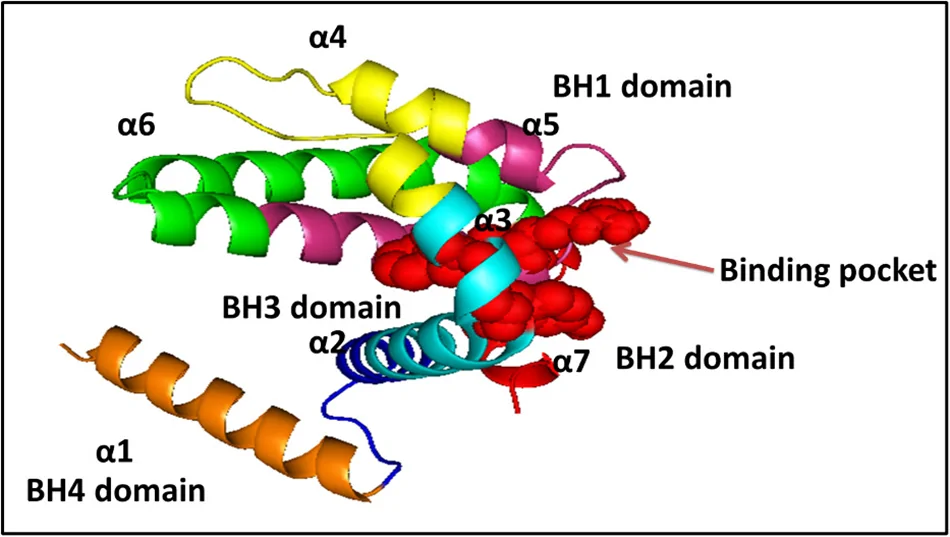
Representative image showing the structure of BCL-XL with its BH domain forming the active site (binding pocket).

Lung cancer is the leading cause of cancer-associated mortality worldwide, primarily due to its late diagnosis and resistance to conventional therapies (1–3). Evasion of apoptosis in cells, or disruption of the apoptotic pathway, is a hallmark of cancer progression, chemoresistance, and tumor survival (4). Apoptosis is a fundamental process for maintaining cellular homeostasis and the cell cycle (5). Apoptosis is mediated by extrinsic and intrinsic pathways, which are regulated by antiapoptotic BCL-2 family members (6). Among them, B-cell lymphoma-extra large (BCL-XL) is a crucial regulator of cellular and metabolic functions (6). BCL-XL is an antiapoptotic protein that plays a key role in regulating the mitochondrial-mediated pathway of apoptosis. It acts by regulating mitochondrial outer membrane permeabilization (6–8). BCL-XL is found to be overexpressed in certain cancer types, such as prostate cancer, leukemia, pancreatic cancer, lung cancer, and breast cancer, a key factor responsible for tumor progression and malignancy (9, 10). The BCL-XL structure consists of eight *α*-helices (*α*1–*α*8) and conserved BCL-2 homology BH1–BH4 domains. The hydrophobic binding pocket formed by BH1–BH3 domains serves as a critical active site for interaction with pro-apoptotic proteins like BAX and BAK, whereas the BH4 domain is involved in antiapoptotic activities (6). Its structure also has C terminal trans membrane domain that helps anchor it to the mitochondrial membrane (11). The BH3 hydrophobic groove has been extensively studied for its interaction with various ligands or drugs to induce apoptosis in cancer cells (5, 12, 13). The representative image of the BCL-XL structure with its BH domains is provided in Figure 1.

Earlier studies have shown that several FDA-approved drugs and BH3 mimetics, particularly Navitoclax (ABT-263), venetoclax (ABT-199), and A-1331852, have been designed to target BCL-XL and induce apoptosis in cancer cells (6, 8, 14, 15). Several clinical trials have shown that these BH3 mimetics downregulate the expression of antiapoptotic proteins (16). However, these BCL-XL inhibitors have encountered clinical limitations, such as dose-limiting thrombocytopenia because platelets rely on BCL-XL for survival, and have limited effects on solid tumors (6, 7, 17). Hence, there is an emergent need to identify alternative approaches or therapeutic agents with improved specificity, outcomes, and fewer side effects.

There is a growing scientific interest and evidence supporting the use of phytocompounds derived from medicinal plants for the treatment of cancer (18–20). These phytocompounds are rich sources of bioactive molecules exhibiting lower toxicity, improved bioavailability, and modulation of multiple targets involved in uncontrolled proliferation (21). Among medicinal plants, *Ailanthus excelsa*, commonly known as “Tree of Heaven,” has drawn our attention due to its wide range of pharmacological properties, including antiinflammatory, antioxidant, and anticancer properties (22–24). In the Chhattisgarh region of India, *A. excelsa* has been traditionally used as a medicine to treat cancer (25). Despite its pharmacological importance, the anticancer potential of the phytoconstituents present in the leaves of *A. excelsa* remain underexplored. Phytochemical analysis of *A. excelsa* by gas chromatography–mass spectrometry (GC–MS) enables the investigation of small-molecule bioactive compounds that can be screened for their ability to bind to the BCL-XL protein, thereby modulating its functions.

In this study, our goal is to identify natural compounds extracted from the chloroform extract of *A. excelsa* that target the antiapoptotic BCL-XL protein. We employed GC–MS to isolate, purify, and characterize 32 phytocompounds from the chloroform extract. Following this, we conducted an absorption, distribution, metabolism, excretion, and toxicity (ADMET) analysis to select the compounds squalene and decane,1,9-bis[(trimethylsilyl)oxy] (aliphatic hydrocarbons), suitable for molecular docking, which show binding affinity toward the BH3-binding pocket of the BCL-XL protein. Then, both compounds were subsequently subjected to molecular dynamics simulations to assess the dynamics and stability of ligand binding to the BCL-XL protein. Decane,1,9-bis[(trimethylsilyl)oxy] is a derivatized organosilicon compound with pharmaceutical properties such as antioxidant, antimicrobial, and anti-inflammatory (26). Squalene is a natural triterpenoid, which is a precursor of cholesterol biosynthesis, and possesses immunomodulatory and chemopreventive properties (27). We validated the anticancer potential of the chloroform extract of *A. excelsa* using an MTT assay in human lung cancer A549 cell lines. This integrative approach not only explains the structure-based interactions between the compounds and BCL-XL but also provides a rationale for plant-based anticancer therapy targeting the apoptosis evasion mechanism.

## Methodology

### Sample collection

Plant specimen *A. excelsa* (commonly known as Tree of Heaven) was purchased from a local market in Delhi, India, where it is traditionally used for medicinal purposes.

### Preparation of the phytochemical extract (PHE)

The fresh leaves of *A. excelsa* were thoroughly washed with water to remove contaminants, shade-dried at room temperature for 10–15 days, and then coarsely powdered for extraction with chloroform in a Soxhlet apparatus for 8 h (22, 28, 29). The extract was filtered and concentrated under reduced pressure using a rotary evaporator and then stored at 4°C in an amber glass container. The resulting dried extract was redissolved in chloroform, and then the phytocompounds were analyzed by GC–MS.

### GC–MS of the chloroform extract of *A. excelsa*

The medicinal plant material was extracted with chloroform using a Soxhlet extractor. The resulting extract was concentrated using a rotary evaporator, and 1–2 µL of the concentrated extract was injected into the GC–MS equipment (Shimadzu GCMS-QP-2010 Plus). The instrument was equipped with an Rtx®-5MS low-bleed column (30 mm × 0.25 mm ID, with a 0.25-µm film thickness). The oven temperature was programmed from 140°C to 280°C at a rate of 5°C/min. The injector temperature was maintained at 270°C, and helium carrier gas was used at a constant flow rate of 1 mL/min. A 0.3 µL sample volume was injected at a pressure of 77.6 kPa, with a purge flow of 3.0 mL/min and a linear velocity of 40.3 cm s^−1^ in split mode (split ratio 10:1). The mass spectra were collected in electron ionization mode at 70 eV and a scan range of m/z 40–600. The obtained mass spectra were analyzed, and the compounds were identified by comparing the spectral data with those in the NIST and Wiley database libraries.

### Computational studies

#### Protein and ligand preparation

The crystal structure of antiapoptotic protein BCL-XL was obtained from the RCSB-Protein Data Bank (PDB ID: 3ZLR). PyMOL2 software was used to clean the protein structure by removing water molecules, attached ligands, and ions bound to the protein. The AutoDock tool (version 1.5.6) was used to prepare the protein for molecular docking. Blind docking was performed using a grid box centered at *X* = 43.633, *Y* = 22.883, and *Z* = 56.787, with box dimensions of 46 × 52 × 60 Å, encompassing the entire BCL-XL protein to enable unbiased exploration of potential binding sites. Polar hydrogen bonds were added, and Gasteiger and Kollman charges were assigned to the protein (30).

Of a total of 32 screened compounds, eight compounds were selected based on their compliance with ADMET properties and Lipinski's Rule of Five, indicating favorable drug-likeness (31, 32). The ADMET parameters, including lipophilicity (logP), hydrogen-bond donors and acceptors, gastrointestinal absorption, and cytotoxicity profile, were evaluated using SwissADME and ADMETlab 2.0 online software. The 3D structures of the selected compounds were retrieved from PubChem using respective compound IDs and downloaded in .sdf format. To prepare the ligand for docking, the Open Babel tool (version 2.3) was used to convert the SDF format to the PDB format (33). Then, the ligand was preprocessed by assigning torsion angles and rotatable bonds using the AutoDock tool (version 1.5.6). Finally, both the processed structures of the protein and the ligand were saved in PDBQT format for further molecular docking analysis (34).

#### Molecular docking

Molecular docking was performed to assess the binding affinity between BCL-XL and the screened compounds. AutoDock Vina (version 1.5.6) was employed for docking, using the Pdbqt files of both the protein and the ligand. The resulting docked complexes with the highest binding energy were analyzed to identify the site or type of interaction between the protein and the ligand (35). Molecular interactions, particularly hydrophobic interactions, within the BCL-XL active site were visualized using LigPlot (version 2.2) and PyMOL2 (36).

#### Molecular dynamics simulations

Simulations were conducted for 100 ns using GROMACS (version 4.0.5) on the two best-docked complex compounds {decane,1,9-bis[(trimethylsilyl)oxy] and squalene} with BCL-XL. In this study, we performed simulations of the docked complex and protein BCL-XL alone. To our knowledge, this is the first report of a simulation of the BCL-XL protein with the selected hydrocarbon compounds. The Amber99 force field was used to create the virtual box, enabling the solvation of the protein–ligand complexes within it (37). The system was inserted into the water box of TIP3P water and neutralized by adding counterions (38). The protonation states of the ionizable group were kept at pH 7. The SHAKE algorithm was utilized to constrain all hydrogen-atom bonds. Electrostatic interactions were computed using the particle mesh Ewald method (39). Prior to the simulation run, the initial geometry of each system was optimized using a steepest descent algorithm with 5,000 iterations. Simulations were conducted under periodic boundary conditions using the NPT ensemble, with temperature and pressure maintained at 300 K and 1 bar via a thermostat and barostat, respectively (40). Finally, MD simulations were run for 100 ns, and the xmgrace command was used to analyze the simulation trajectories.

### Cell culture

Human lung cancer cell line (A549) was procured from the National Center for Cell Science (NCCS), Pune, and cells were cultured in RPMI-1640 media supplemented with 10% fetal bovine serum and 1% penicillin–streptomycin. The cell culture was maintained at 37°C in a humidified incubator with 5% CO_2_ (41, 42). The A549 human lung cancer cell line was maintained according to the supplier's recommended protocol and used at low passage number for all experiments.

### MTT assay

The cytotoxic effects of bioactive compounds were evaluated using the MTT assay on lung cancer cell line (A549). In a sterile 96-well plate, cells were seeded at a density of 1 × 10^4^ cells per well and incubated to adhere overnight. After 24 h, cells were treated with different concentrations of the extract (31.25–500 μg/mL) prepared in DMSO. Untreated cells were used as the negative control, and the DMSO-only well was used to rule out solvent effects on cancer cells. After different incubation periods, 20 µL of MTT solution was added to each well and further incubated for 3–4 h at 37°C. After incubation, 150 µL of DMSO was added to each well to dissolve the crystals formed by viable cells. The absorbance was measured at 570 nm using a microplate reader (BioRad iMark). The IC_50_ value was calculated using the GraphPad Prism (version 8.0). The MTT experiments were performed in three independent biological replicates. Data are presented as mean ± standard deviation. A *p*-value of <0.05 was considered statistically significant.

## Results

### GC–MS analysis

**Figure 2:**
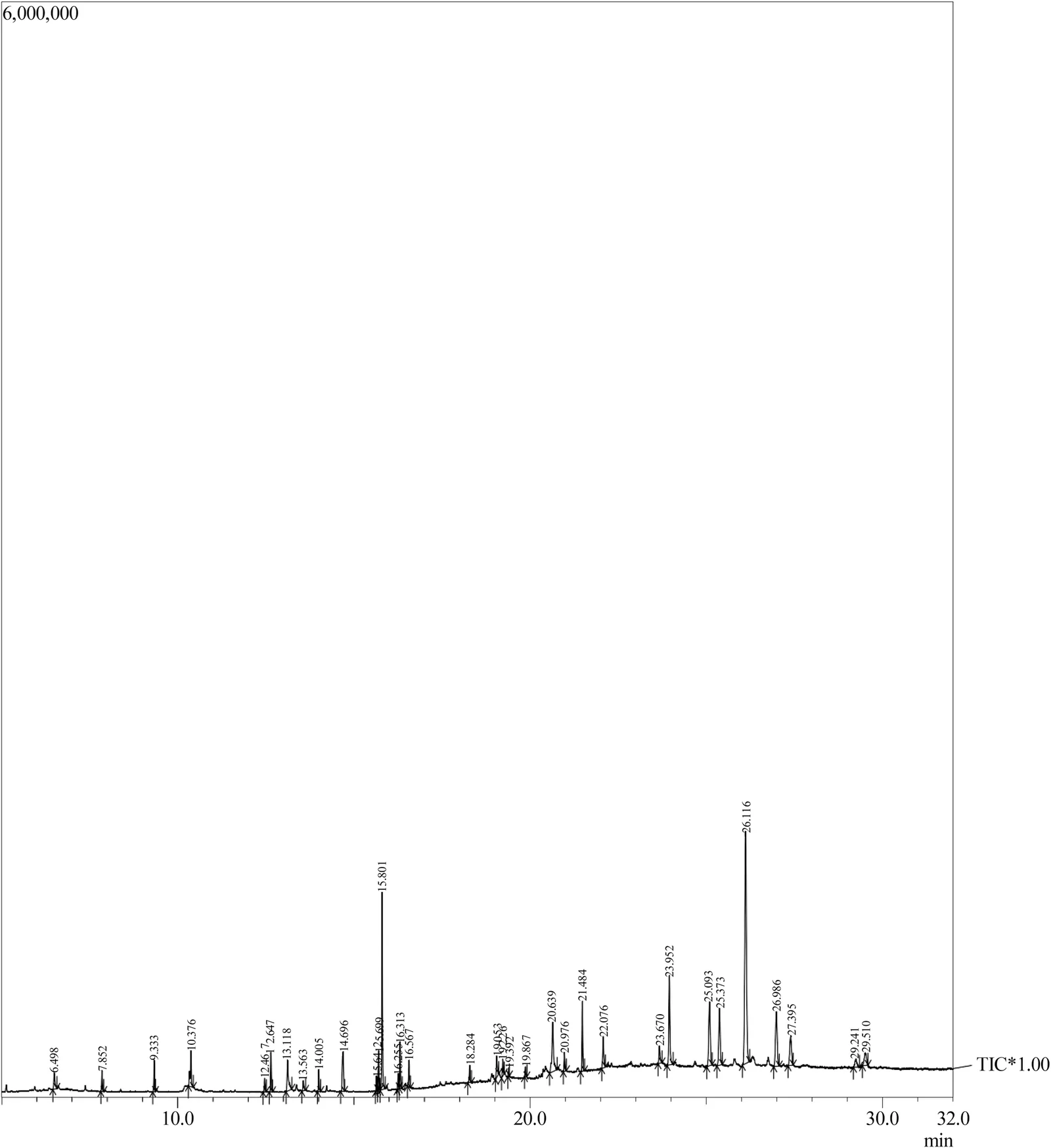
GC–MS chromatogram of the chloroform extract of *A. excelsa* showing the peak value of the presence of different compounds at different retention times.

The phytochemical constituents present in the chloroform extract of *A. excelsa* leaves were analyzed by GC–MS. A total of 32 bioactive compounds with different *m*/*z* ratios, molecular weights, nature of compounds, and retention times are listed in Table 1. Chromatograms of all the phyto-components are shown in Figure 2. The constituents identified in the chloroform extract of *A. excelsa* leaves included neophyttadiene (2.17), lupeol (3.16%), squalene (3.27%), 1-hentetracontanol (4.95%), lup-20(29)-en-3-one (5.51%), ergost-5-en-3-ol (6.01%), vitamin E (6.19%), phytol (9.18%), and stigmast-5-en-3-ol (23.36%). These compounds in the extract are well known for their anticancer, anti-inflammatory, and antioxidant properties, which contribute to the biological activity. This finding demonstrates the presence of a bioactive, compound-rich source in the extract of *A. excelsa*, which may contribute to its traditional medicinal use in cancer treatment.

**Table 1:** Retention time and relative peak area (%) of phytoconstituents identified in the chloroform extract of *A. excelsa*.

Peak#	Retention time	Area	Area%	Name
1	6.498	226118	1.24	1-Allyl-4-methoxybenzene
2	7.852	170021	0.93	1-Tetradecene
3	9.333	249676	1.37	Phenol, 2,4-bis(1,1-dimethylethyl)-
4	10.376	467905	2.57	9-Eicosene, (E)-
5	12.467	127252	0.70	6-Hydroxy-4,4,7a-trimethyl-5,6,7,7a-tetrahydrobenzofuran-
6	12.647	329279	1.81	1-nonadecene
7	13.118	493627	2.71	Neophytadiene
8	13.563	122463	0.67	3,7,11,15-Tetramethyl-2-hexadecen-1-ol
9	14.005	175815	0.96	Hexadecanoic acid, methyl ester
10	15.642	111497	0.61	Oxacycloheptadec-8-en-2-one, (8Z)-
11	15.699	328034	1.80	9,12,15-Octadecatrienoic acid, methyl ester, (Z,Z,Z)-
12	15.801	1674889	9.18	Phytol
13	16.255	117247	0.64	11,14-Eicosadienoic acid
14	16.313	367862	2.02	Ethyl (9z,12z)-9,12-octadecadienoate
15	16.567	246749	1.35	Pentafluoropropionic acid, pentadecyl ester
16	18.284	157934	0.87	Octacosanol
17	19.053	256273	1.41	Decane,1,9-bis[(trimethylsilyl)oxy]
18	19.392	65208	0.36	1,2-Benzenedicarboxylic acid, ditridecyl
19	19.867	90250	0.49	Octacosyl heptafluorobutyrate
20	20.639	902416	4.95	1-Hentetracontanol
21	20.976	171927	0.94	1,4-Benzenedicarboxylic acid, bis(2-ethylhexyl) ester
22	21.484	596041	3.27	Squalene
23	22.076	376568	2.06	Dotriacontyl pentafluoropropionate
24	23.670	236428	1.30	Octacosanol
25	23.952	1129737	6.19	Vitamin E
26	25.093	1095395	6.01	Ergost-5-en-3-ol, (3.beta.,24r)-
27	25.373	887694	4.87	Stigmasta-5,22-dien-3-ol
28	26.116	4259874	23.36	Stigmast-5-en-3-ol, (3.beta.)-
29	26.986	1004529	5.51	Lup-20 (29)-en-3-one
30	27.395	576893	3.16	Lupeol
31	29.241	176991	0.97	17-[5-(1-Hydroxy-1-methyl-ethyl)-2-methyl-t
32	29.510	324193	1.78	Phytol stearate
		18236281	100.00	

### ADMET analysis of *A. excelsa* extract-derived compounds and molecular docking

**Figure 3:**
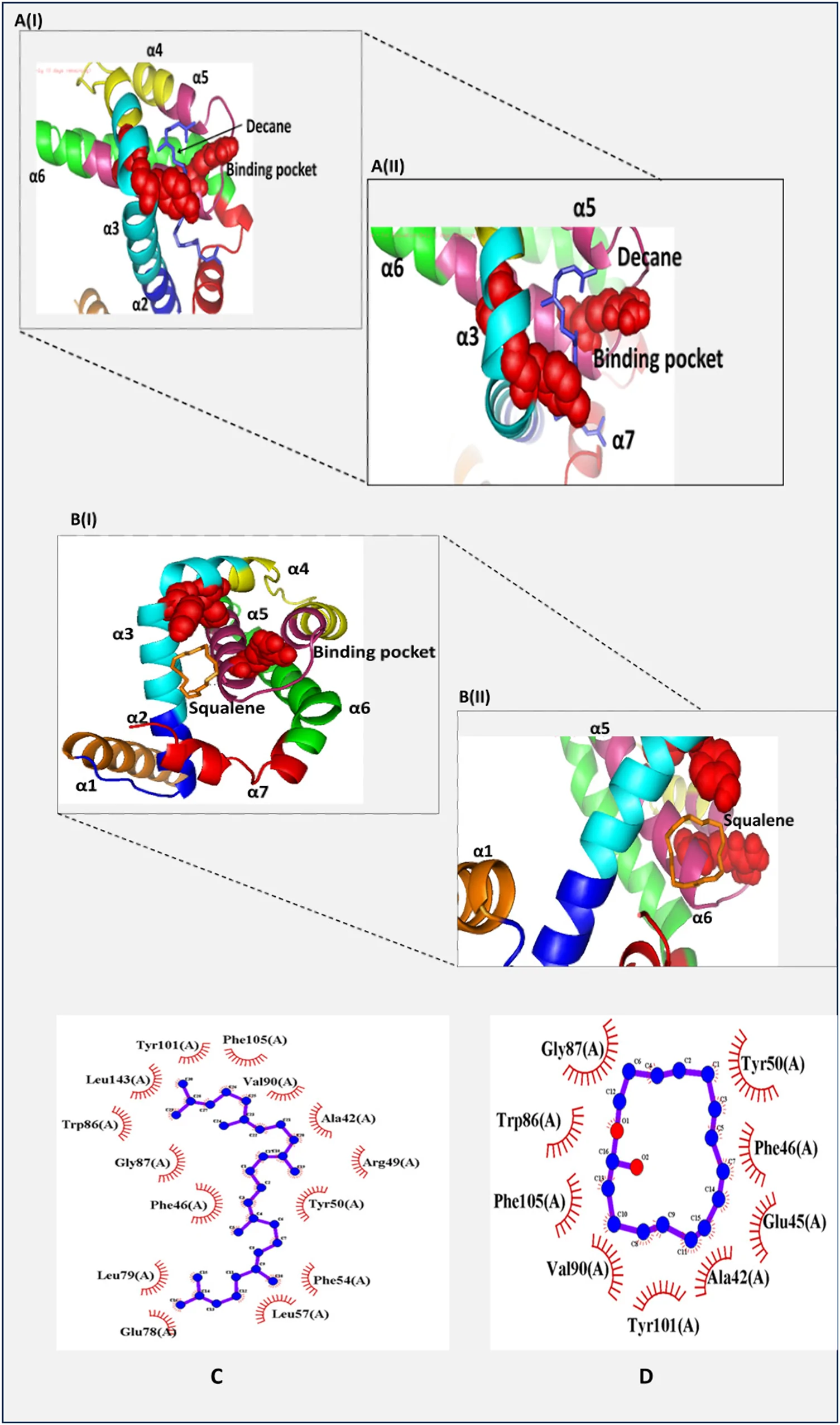
Representation of the docked complex of decane,1,9-bis[(trimethylsilyl)oxy] and squalene with BCL-XL. **(A-I)** 3D structure of BCL-XL with docked ligand decane,1,9-bis[(trimethylsilyl)oxy], as shown in blue stick in the binding pocket; **(B-I)** docked complex of BCL-XL with squalene (orange stick). **(A,B-II)** zoomed view; **(C,D)** LigPlot 2D diagram showing the interactions between the ligands—decane,1,9-bis[(trimethylsilyl)oxy] and squalene—and hydrophobic residues of BCL-XL.

The chloroform extract of medicinal plants contains various compounds, each with different chemical properties. A total of 32 compounds from this extract were subjected to ADMET analysis to evaluate their pharmacokinetic profiles and oral bioavailability (Supplementary Table S1). Of these compounds, eight met the criteria of Lipinski's Rule of Five (Supplementary Table S2), which considers molecular weight below 500 Da, logP, the number of hydrogen bond donors and acceptors, and topological polar surface area (32, 43). The filtered compounds were computationally docked against the antiapoptotic protein BCL-XL (PDB ID: 3ZLR) using AutoDock Vina (version 1.5.6). Among them, decane,1,9-bis[(trimethylsilyl)oxy] (a saturated hydrocarbon) and squalene (a polyunsaturated hydrocarbon) showed lower binding affinities toward BCL-XL, with docking scores of −8.6 and −7.6 kcal/mol, respectively (Supplementary Table S2). Further interaction analysis revealed that both compounds interact with the hydrophobic groove of the BH3 domain of the BCL-XL protein, which is crucial for their antiapoptotic activity (Figures 3A,B). The LigPlot (version 2.2) analysis showed that common residues at Tyr101 (*α*2), Phe105 (*α*2–*α*3 junction), and Ala 42 (*α*5) present in these helices form the hydrophobic groove or binding pocket for squalene and decane,1,9-bis[(trimethylsilyl)oxy] interaction (Figures 3C,D). No significant polar hydrogen bonds and electrostatic interactions were found between decane,1,9-bis[(trimethylsilyl)oxy] or squalene and the BCL-XL protein. This result indicates that the major driving force stabilizing the compound’s interaction with BCL-XL is hydrophobic. This interaction closely mimics that observed with the known BCL-XL inhibitor, supporting their potential as a therapeutic lead for anticancer therapy.

### Molecular dynamics simulations

#### Root mean square deviation (RMSD), root mean square fluctuation (RMSF), radius of gyration (Rg), and solvent accessibility surface area parameters

**Figure 4:**
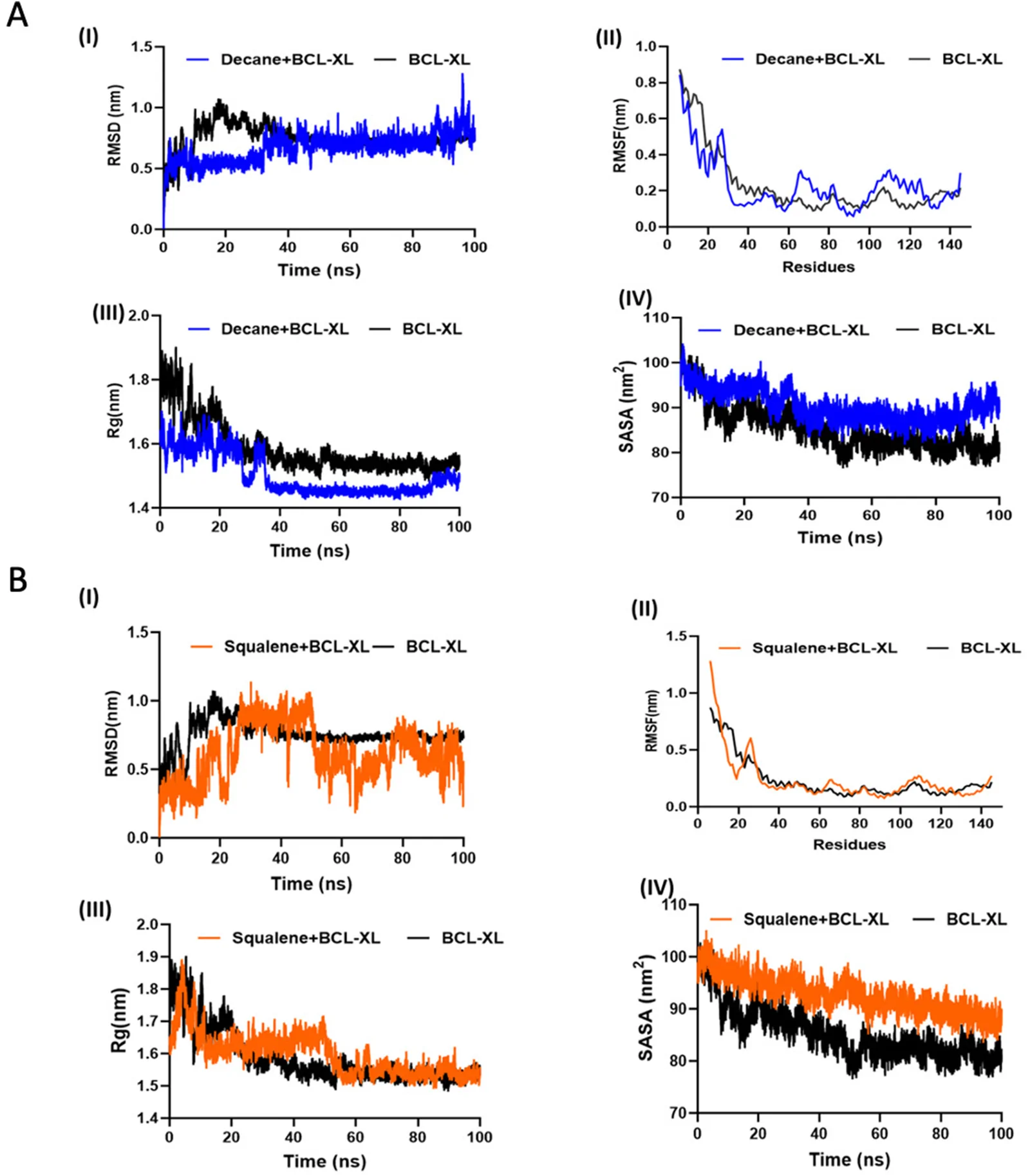
**(A)** representative image depicting the MD simulation of decane,1,9-bis[(trimethylsilyl)oxy] with the BCL-XL protein: **(I)** RMSD, **(II)** RMSF, **(III)** Rg, and **(IV)** SASA; BCL-XL–decane,1,9-bis[(trimethylsilyl)oxy] (blue), BCL-XL (black). **(B)** Representative image depicting the MD simulation of squalene with the BCL-XL protein: **(I)** RMSD, **(II)** RMSF, **(III)** Rg, and **(IV)** SASA; BCL-XL–squalene (orange), BCL-XL (black).

The RMSD analysis shows that the BCL-XL–squalene complex exhibited lower deviation in the initial phase of simulations (0–20 ns) than the BCL-XL protein alone, followed by consistent stabilization [Figure 4B (I)]. However, the complex shows higher fluctuation, indicating that the binding of squalene induces the conformational changes but does not destabilize the protein structure [Figure 4B (I)]. In contrast, the decane,1,9-bis[(trimethylsilyl)oxy]–BCL-XL complex shows lower fluctuation than the BCL-XL protein alone and then maintains the structural conformation throughout the 100 ns simulations [Figure 4A (I)]. This stabilization in conformation is further supported by a low radius of gyration (Rg) value (1.45–1.55 nm), depicting its compactness [Figure 4A (III)]. Meanwhile, the Rg value of the complex with squalene shows minor fluctuation, indicating a less pronounced effect on protein structure [Figure 4B (III)].

**Figure 5:**
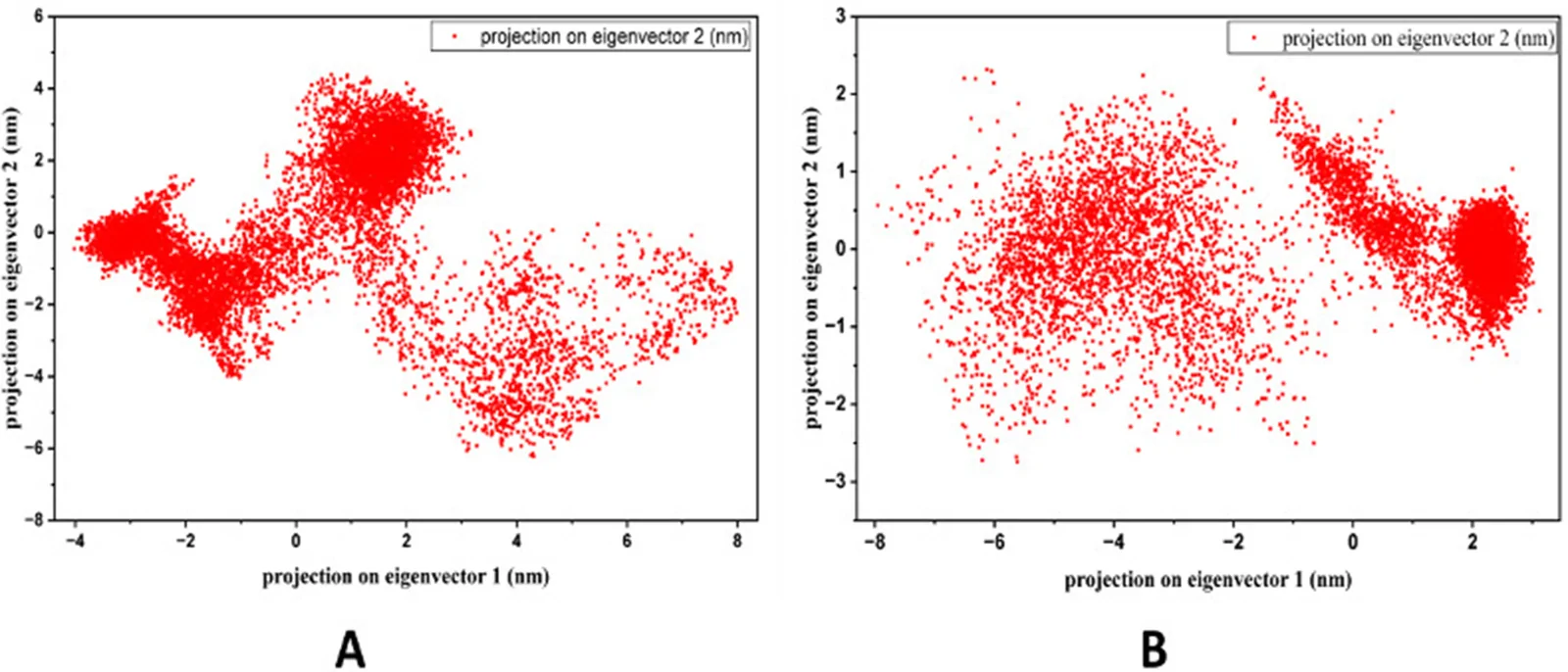
Principal component analysis (PCA) of the BCL-XL protein in complex with **(A)** squalene and **(B)** decane,1,9-bis[(trimethylsilyl)oxy].

The RMSF analysis of the decane,1,9-bis[(trimethylsilyl)oxy]–BCL-XL complex [Figure 4A (II)] exhibited higher fluctuation in the regions spanning amino acid residues 60–80 and 100–120 in the BH3 domain, indicating that decane,1,9-bis[(trimethylsilyl)oxy] binding reduces the flexibility, especially in the loop regions, compared to the native BCL-XL protein. This reduction in flexibility is consistent with the principal component analysis (PCA) result (Figure 5B). The RMSF graph of the squalene–BCL-XL complex [Figure 4B (II)] shows minimal fluctuation compared to the native BCL-XL protein alone. The solvent accessibility surface area (SASA) plot shows that complexes of the BCL-XL protein with both squalene and decane,1,9-bis[(trimethylsilyl)oxy] showed higher SASA values, indicating the high accessibility of the protein structure to the solvent [Figures 4A,B (IV)].

### Principal component analysis and free energy landscape

PCA was conducted to describe the most prominent motions of BCL-XL in complex with squalene (Figure 5A) and decane,1,9-bis[(trimethylsilyl)oxy] (Figure 5B). The first two eigenvectors (PC1 and PC2) were utilized to project the essential dynamics.

The PCA projection of the BCL-XL–squalene complex reveals several well-distinguishable clusters scattered over the conformational space, suggesting that it samples a wide conformational landscape with high structural fluctuations. Several clusters indicate switching among various metastable states, which suggests that squalene binding imposes large-scale flexibility and conformational remodeling in BCL-XL (Figure 5A). Conversely, the PCA projection of the BCL-XL–decane,1,9-bis[(trimethylsilyl)oxy] complex shows a narrower distribution with smaller, denser clusters. The conformational sampling is limited, indicating that decane,1,9-bis[(trimethylsilyl)oxy] binding stabilizes BCL-XL in fewer conformations and restricts its mobility (Figure 5B).

**Figure 6:**
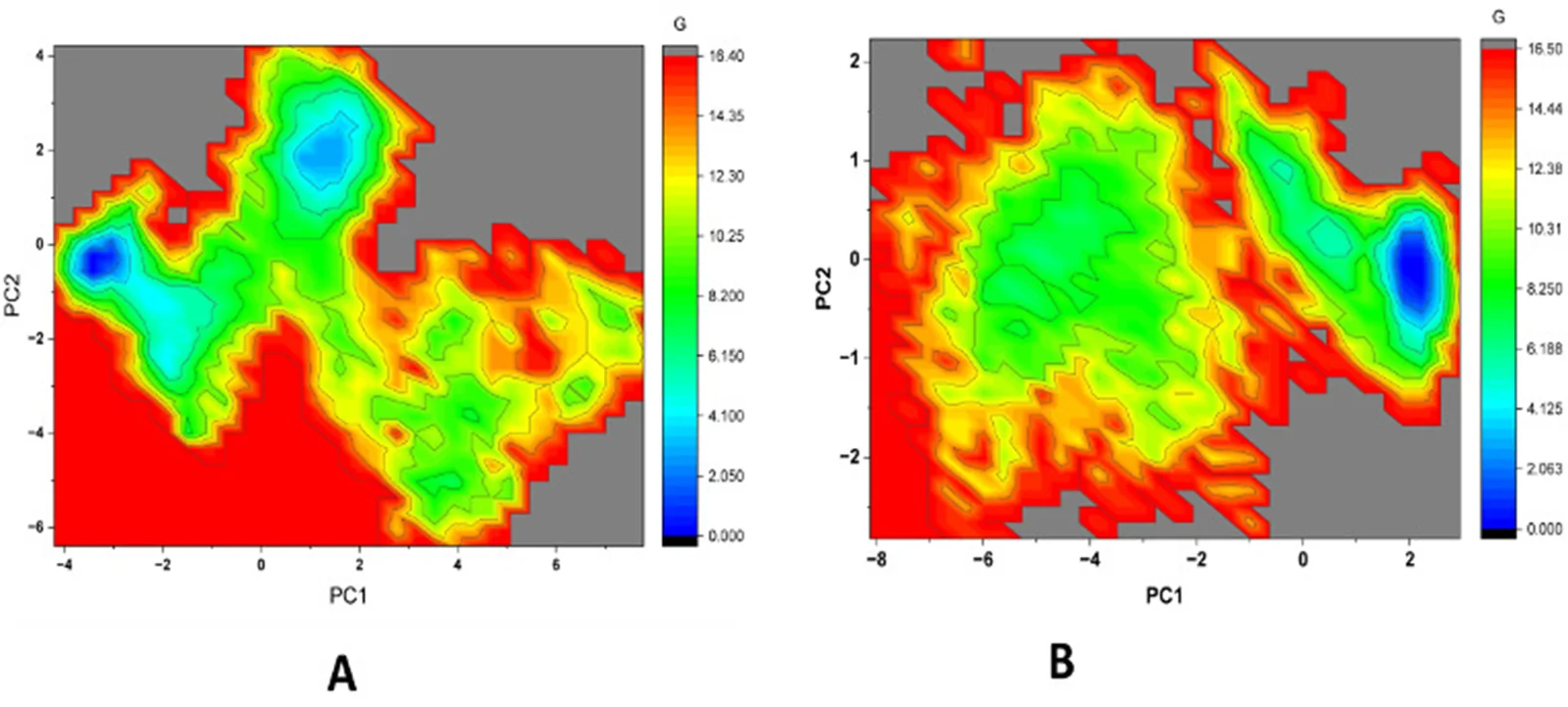
Free energy landscape (FEL) of the BCL-XL protein in complex with **(A)** squalene and **(B)** decane,1,9-bis[(trimethylsilyl)oxy] by projecting the molecular dynamics trajectories onto the first two principal components (PC1 and PC2). The color scheme represents the Gibbs free energy (kcal/mol), and the color bar ranges between 0 and 16.5.

The free-energy landscape (FEL) of BCL-XL in complex with both ligands exhibited dramatically different conformational preferences (Figures 6A,B). When bound to squalene, the protein explored a landscape with many low-energy minima (blue to cyan region) scattered throughout the PC1–PC2 space (Figure 6A). The separated minima correspond to metastable conformations and are uneven, with several intermediate energy basins (green to yellow), implying that squalene binding increases the conformational heterogeneity of BCL-XL.

On the other hand, the FEL of the BCL-XL–decane,1,9-bis[(trimethylsilyl)oxy] complex showed a relatively smoother topology, which was characterized by a large central basin and one deep-energy well (intense blue on the right side of the plot) (Figure 6B). This reveals that decane,1,9-bis[(trimethylsilyl)oxy] stabilizes fewer energetically favorable conformations of BCL-XL, thereby limiting conformational fluctuations.

### Cytotoxic effect on lung cancer cell line (A549)

The chloroform extract of *A. excelsa* was evaluated for its cytotoxic effect on lung cancer cell line (A549) using the MTT assay. Cells were treated for 24–48 h with increasing concentrations of the extract. We observed a dose-dependent decrease in cell viability in lung cancer cells, representing cytotoxic response. The plant extract with an IC50 value of 61.27 µg/mL showsed a significant decrease in cell viability. At high dosages, the extract of *A. excelsa* induced nearly 100% cell death in lung cancer cells. These results indicate the presence of bioactive compounds with anticancer properties in the leaves of *A. excelsa*.

## Discussion

Apoptosis resistance, a regulated mechanism, leads to the overexpression of BCL-2, an antiapoptotic protein family member, which is considered a hallmark and is responsible for causing cancer-related mortality worldwide (6, 9, 44). BCL-XL, a member of the BCL2 family, inhibits the mitochondrial outer membrane permeabilization, prevents the release of cytochrome C, and subsequently hinders caspase activation (45). BCL-XL is identified as a target to restore apoptotic potential, representing a promising therapeutic approach (46). However, BCL-XL, including FDA-approved drugs and BCL-XL mimetics (6, 47), has shown limitations due to clinical challenges, resistance, toxicity, and side effects, which emphasize the need for a safer alternative approach that selectively inhibits the BCL-XL protein (48).

In this study, we investigated bioactive phytocompounds extracted from *A. excelsa* as potential BCL-XL inhibitors through an integrated in silico and *in vitro* approach. The GC–MS analysis of the chloroform leaf extract of *A. excelsa* showed the presence of 32 bioactive compounds, including terpenoids, phenolics, sterols, hydrocarbons, and esters (Table 1; Figure 2). These compounds have been associated with anti-inflammatory, antioxidant, and cytotoxic activities. The identified components with peak area percentages were stigmasterol, lupeol, vitamin E, ergosterol derivatives, squalene, phytol, and neophytadiene. Eight compounds were selected based on their favorable physicochemical properties following ADMET profiling (Supplementary Table S2). The selected compounds, including decane,1,9-bis[(trimethylsilyl)oxy] derivatives, squalene, stigmasterol, and lupeol, were found to obey Lipinski's Rule of Five, which is beneficial for drug likeness and oral bioavailability. Among these, decane,1,9-bis[(trimethylsilyl)oxy] (−8.6 kcal/mol) and squalene (−7.6 kcal/mol) showed the most favorable docking interactions with BCL-XL.

Although decane,1,9-bis[(trimethylsilyl)oxy] has been less explored as a phytochemical with anticancer properties, similar trimethylsilyl compounds have been identified in GC–MS analysis of several medicinal plants and have been associated with biochemical properties. Previous research has reported that the detection of these trimethylsilyl-derived compounds in plant extracts suggests that these compounds may contribute to biological activity (26, 49). In the present study, decane,1,9-bis[(trimethylsilyl)oxy] was prioritized not only because it was detected in GC–MS analysis of the chloroform extract of *A. excelsa* but also because it exhibited ADMET properties and the highest binding affinity toward the BCL-XL protein. However, further validation is required to establish its natural occurrence and contribution to the anticancer activity of *A. excelsa*.

Docking analysis shows that decane,1,9-bis[(trimethylsilyl)oxy] and squalene bind to the BH3-binding groove of BCL-XL, which could be essential for their antiapoptotic activity. These compounds form stable hydrophobic interactions with key residues Tyr101, Phe105, and Ala42, along with Gly87, Trp86, Val90, Phe46, Tyr50, and Glu45 in the BH3 domain, which is critical for inhibiting the pro-apoptotic protein (Figures 3A–D). Additional molecular dynamics simulations confirmed the dynamic stability of the protein–ligand complexes (Figures 4, 5). The RMSD and Rg analyses indicated that decane,1,9-bis[(trimethylsilyl)oxy]-bound BCL-XL maintained higher compactness and lower flexibility [Figure 4A (I, III)]. However, squalene binding does not produce any instability in conformational dynamics of the protein structure [Figure 4B (I, III)]. The RMSF plot of squalene suggested that lower fluctuations in certain regions restrict the flexibility of the binding or active-site BH3 domain of the BCL-XL protein [Figure 4B (II)].

Together, the PCA and FEL (Figures 5, 6) analyses exhibited that squalene allows conformational flexibility and dynamic structural rearrangements of BCL-XL, enabling the protein to form metastable states, while decane,1,9-bis[(trimethylsilyl)oxy] limits the conformational space and stabilizes BCL-XL into fewer conformational states (Figures 5B, 6B). The simulation results indicated that the enhanced flexibility caused by squalene can destabilize the BH3-binding groove and reduce the antiapoptotic ability of BCL-XL, while the stabilizing influence of decane,1,9-bis[(trimethylsilyl)oxy] indicates reduced modulatory ability.

The MTT assay further confirmed that *A. excelsa* extract exhibited cytotoxicity (IC_50_ 61.27 µg/mL) against A549 lung cancer cells, suggesting its potential effectiveness in treating lung cancer. However, the observed cytotoxicity cannot be directly attributed to decane,1,9-bis[(trimethylsilyl)oxy], squalene, or any individual compound. The extract contains multiple phytochemicals that contribute individually or synergistically to the observed cytotoxic effect. Therefore, the proposed involvement of decane,1,9-bis[(trimethylsilyl)oxy] and squalene is based on computational prediction and should be considered a hypothesis rather than a demonstrated mechanistic conclusion. Further studies involving compound isolation, structural confirmation, and individual biological inference are necessary to validate the specific contribution of these compounds to the anticancer activity of *A. excelsa*.

Although the chloroform extract of *A. excelsa* showed significant cytotoxicity against the lung cancer cell line A549 using the MTT assay, the present study is limited by the lack of direct mechanistic validation of apoptosis and BCL-XL inhibition. Therefore, additional studies, including the apoptosis assay, caspase activity assay, and western blotting of apoptosis-related proteins, are required to validate the involvement of apoptotic molecular pathways and the modulation of the BCL-XL protein. Overall, our results indicate that phytocompounds derived from *A. excelsa* could specifically target and inhibit the antiapoptotic action of BCL-XL, with promising anticancer activity. The integrated computational analysis and *in-vitro* cytotoxicity results support the therapeutic potential. This combined approach signifies the therapeutic potential of phytochemicals as either adjuvant or standalone therapies for lung cancer treatment. Further extensive *in vitro* apoptosis experiments, western blot analysis of BCL-XL expression, and *in vivo* studies will be needed to establish the mechanistic role and therapeutic potential of phytocompounds derived from *A. excelsa*.

## Data Availability

The original contributions presented in the study are included in the article/Supplementary Material, further inquiries can be directed to the corresponding author.
