# Supplementary material for: Targeting BCL-XL-mediated apoptotic resistance in lung cancer: in silico and in vitro identification of bioactive inhibitors from Ailanthus excelsa chloroform extract
Source: Front Med (Lausanne). 2026 Sep 9;13:1925157. doi: 10.3389/fmed.2026.1925157 (PMC13597438; doi:10.3389/fmed.2026.1925157)
Supplement: Supplementary file 1 [file Table1.docx]

**Targeting anti-apoptotic BCL-XL protein with Squalene and Decane phytocompounds from chloroform extract of leaves of *Ailanthis excelsa*: A computational and experimental approach against lung cancer**

**Table S1** represent the list of compounds analyzed by SwissADME, including their LIPINSKI violations, HBD (Hydrogen bond donor), HBA ( Hydrogen bond Acceptor), Log P represent the lipophilicity of compounds; M.WT (Molecular Weight)

| S.No. | Compound name | CID | M.WT | LogP | HBD | HBA | No. of Violation | G.I absorption | Bioavailability |
| --- | --- | --- | --- | --- | --- | --- | --- | --- | --- |
| 1. | 1-Allyl-4-methoxybenzene | 8815 | 148.2 | 2.47 | 0 | 1 | 0 | High | 0.55 |
| 2. | 1-Tetradecene | 14260 | 196.37 | 4.1 | 0 | 0 | 1 | Low | 0.55 |
| 3. | 2-hydroxy-1-(hydroxymethyl)ethyl ester | 985 | 256.42 | 3.85 | 1 | 2 | 1 | High | 0.85 |
| 4. | 1,2-benzenedicarboxylic acid | 1017 | 166.13 | 0.6 | 2 | 4 | 0 | High | 0.85 |
| 5. | Vitamin E | 2117 | 472.74 | 5.81 | 0 | 3 | 1 | Low | 0.55 |
| 6. | 1,4-Benzenedicarboxylic acid | 7489 | 166.13 | 0.74 | 2 | 4 | 0 | High | 0.85 |
| 7. | Hexadecanoic acid | 8181 | 270.45 | 4.41 | 0 | 2 | 1 | High | 0.55 |
| 8. | Neophytadiene | 10446 | 278.52 | 4.32 | 0 | 0 | 1 | Low | 0.55 |
| 9. | 6-Hydroxy-4,4,7a-trimethyl-5,6,7,7a-tetrahydrobenzofuran- | 14334 | 196.24 | 1.88 | 1 | 3 | 0 | High | 0.55 |
| 10. | Stigmast-5-en-3-ol, (3.beta.)- | 222284 | 414.71 | 5.05 | 1 | 1 | 1 | Low | 0.55 |
| 11. | Lupeol | 259846 | 426.72 | 4.72 | 1 | 1 | 1 | Low | 0.55 |
| 12. | Decane, | 575943 | 318.64 | 5.15 | 0 | 2 | 0 | High | 0.55 |
| 13. | Squalene | 638072 | 410.72 | 5.15 | 0 | 0 | 0 | High | 0.55 |
| 14. | Phytol | 5280435 | 296.53 | 4.85 | 1 | 1 | 1 | Low | 0.55 |
| 15. | Stigmasta-5,22-dien-3-ol | 5280794 | 412.69 | 5.08 | 1 | 1 | 1 | Low | 0.55 |
| 16. | 9,12,15-Octadecatrienoic acid | 5319706 | 292.46 | 5.08 | 0 | 2 | 1 | Low | 0.55 |
| 17. | Oxacycloheptadec-8-en-2-one | 5365703 | 252.39 | 3.31 | 0 | 2 | 0 | High | 0.55 |
| 18. | 3,7,11,15-Tetramethyl-2-hexadecen-1-ol | 5366244 | 296.53 | 4.83 | 1 | 1 | 1 | Low | 0.55 |
| 19. | Phenol, 2,4-bis(1,1-dimethylethyl)- | 7311 | 206.32 | 3.08 | 1 | 1 | 0 | High | 0.55 |
| 20. | 1-Hentetracontanol | 38627 | 593.11 | 10.31 | 1 | 1 | 2 | Low | 0.17 |
| 21. | Octacosanol | 68406 | 410.76 | 7.2 | 1 | 1 | 1 | Low | 0.55 |
| 22. | Lup-20(29)-en-3-one | 323075 | 424.7 | 4.56 | 0 | 1 | 1 | Low | 0.55 |
| 23. | Pentafluoropropionic acid | 537282 | 374.43 | 4.88 | 0 | 7 | 1 | Low | 0.55 |
| 24. | 17-[5-(1-hydroxy-1-methyl-ethyl)-2-methyl-t | 588518 | 604.81 | 3.93 | 2 | 8 | 2 | Low | 0.56 |
| 25. | 9-Eicosene | 545762 | 280.53 | 5.5 | 0 | 0 | 1 | Low | 0.55 |
| 26. | 11,14-Eicosadienoic acid | 5282805 | 308.5 | 4.41 | 1 | 2 | 1 | High | 0.85 |
| 27. | Octacosyl heptafluorobutyrate | 91693314 | 612.88 | 8.83 | 0 | 7 | 2 | Low | 0.17 |
| 28. | Ergost-5-en-3-ol | 6428659 | 400.68 | 4.96 | 1 | 1 | 1 | Low | 0.55 |
| 29. | Phytyl stearate | 14486562 | 562.99 | 8.72 | 0 | 2 | 2 | Low | 0.17 |
| 30. | Ethyl (9z,12z)-9,12-octadecadienoate | 20833831 | 423.59 | 8.72 | 0 | 5 | 2 | Low | 0.17 |
| 31. | Dotriacontyl pentafluoropropionate | 91692955 | 606.78 | 8.29 | 0 | 9 | 2 | Low | 0.17 |
| 32. | 1-nonadecene | 29075 | 266.5 | 5.28 | 0 | 0 | 1 | Low | 0.55 |

**Table S2** enlisted compounds that comply with Lipinski’s rule of Five and represent their binding affinity with BCL-XL protein.

| S.No | Compound | Docking score Kcal/mol |
| --- | --- | --- |
| 1. | Decane | -8.6 |
| 2. | Squalene | -7.6 |
| 3. | Oxacycloheptadec-8-en-2-one | -6.1 |
| 4. | Phenol, 2,4-bis(1,1-dimethylethyl)- | -6.1 |
| 5. | 1,4-Benzenedicarboxylic acid | -6.0 |
| 6. | 6-Hydroxy-4,4,7a-trimethyl-5,6,7,7a-tetrahydrobenzofuran- | -5.8 |
| 7. | 1,2-benzenedicarboxylic acid | -5.6 |
| 8. | 1-Allyl-4-methoxybenzene | -5.3 |
